# *GmEXLB1*, a Soybean Expansin-Like B Gene, Alters Root Architecture to Improve Phosphorus Acquisition in *Arabidopsis*

**DOI:** 10.3389/fpls.2019.00808

**Published:** 2019-07-05

**Authors:** Youbin Kong, Bing Wang, Hui Du, Wenlong Li, XiHuan Li, Caiying Zhang

**Affiliations:** North China Key Laboratory for Germplasm Resources of Education Ministry, College of Agronomy, Hebei Agricultural University, Baoding, China

**Keywords:** expansin-like B, root architecture, low phosphorus, phosphorus acquisition and utilization, soybean

## Abstract

Expansins comprise four subfamilies, α-expansin (EXPA), β-expansin (EXPB), expansin-like A (EXLA), and expansin-like B (EXLB), which are involved in the regulation of root development and growth under abiotic stress. To date, few EXLB genes have been shown to respond to low phosphorus (P) in plants. In this study, we identified an EXLB gene, *GmEXLB1*, by analyzing the transcription profiles of GmEXLBs in soybean. Quantitative analysis showed that *GmEXLB1* was expressed and induced in the lateral roots of soybean under low P conditions. The observation of β-glucuronidase staining in transgenic *Arabidopsis* suggested that *GmEXLB1* might be associated with lateral root emergence. *GmEXLB1* overexpression altered the root architecture of transgenic *Arabidopsis* by increasing the number and length of lateral roots and the length of primary roots under low P conditions. Additionally, the length of the elongation zone and the average cell length in the elongation zone were increased in transgenic *Arabidopsis*. Increases in biomass and P content suggested that *GmEXLB1* overexpression enhanced P acquisition in *Arabidopsis*. Overall, we conclude that *GmEXLB1* expression is induced in soybean under low P conditions, and the overexpression of *GmEXLB1* improves P acquisition by regulating root elongation and architecture in *Arabidopsis*, which provides a possible direction for research of the function of this gene in soybean.

## Introduction

Phosphorus (P) is an essential macronutrient for plant growth and development (Vance, 2001; Schachtman and Shin, 2007). Inorganic phosphate (P_i_) is the main form of P in soil that is taken up by plants. Owing to its chemical properties, P_i_ forms organic matter or is bound to iron and aluminum in soil (Chiou and Lin, 2011). Thus, the concentration of P_i_ is far below the level required for plant growth. To ensure crop productivity, farmers apply P_i_ fertilizers produced from nonrenewable rock phosphate (Lopez-Arredondo et al., 2014). Many applications of P_i_ fertilizers cause soil degradation and water eutrophication (Gilbert, 2009). Therefore, improving P_i_ absorption and utilization efficiency in crops is important for sustainable agriculture (Song et al., 2014).

The root is the major organ that has adapted to acquire water and nutrition from the soil (Petricka et al., 2012). The root architecture is influenced by environmental factors, including the levels of phosphorus, nitrogen, and iron and water starvation. In the process of long-term evolution, plants have developed a series of response mechanisms to cope with low P that involve changing the root configuration *via* changes in the lateral roots and root hairs (Gutierrez-Alanis et al., 2018). Some studies have demonstrated that the number and length of lateral roots significantly increase in *Arabidopsis* under conditions of low P (Ticconi et al., 2009; Niu et al., 2013; Kellermeier et al., 2014). In addition, high-P efficiency soybean varieties can also improve P uptake by changing root morphology and structure under conditions of low P (Li et al., 2005; Liang et al., 2010). However, the molecular mechanisms underlying alterations in root architecture in response to low P remain unclear.

Expansins are known to be involved in the extension and relaxation of cell walls and are able to regulate the development of the root system (Cosgrove, 2000, 2015; Li et al., 2003; Sampedro and Cosgrove, 2005). Many expansin genes have been identified in plant species, including *Arabidopsis* (Sampedro et al., 2005), rice (Sampedro et al., 2005; Che et al., 2016), maize (Zhang et al., 2014), soybean (Zhu et al., 2014), wheat (Li et al., 2016), and peanut (Guimaraes et al., 2017). Protein structural and genome-wide studies have revealed that expansins comprise four subfamilies (Cosgrove, 2015): α-expansin (EXPA), β-expansin (EXPB), expansin-like A (EXLA), and expansin-like B (EXLB). To date, the majority of expansin genes identified experimentally have been EXPAs and EXPBs. Two *Arabidopsis* expansin genes, *AtEXP7* and *AtEXP18*, have been closely linked to root hair initiation (Cho and Cosgrove, 2002). Similarly, *HvEXPB1* was found to be root-specific and associated with root hair formation in barley (Kwasniewski and Szarejko, 2006). Additionally, the expression of expansin genes in roots is induced by many abiotic stresses, such as phosphorus deficiency (Guo et al., 2011), drought (Wu et al., 2001; He et al., 2015), and high salinity (Chen et al., 2017). Transgenic *Arabidopsis* plants overexpressing *RhEXPA4* exhibited enhanced salt tolerance by increasing the number of lateral roots and the leaf chlorophyll content after salt treatment (Lü et al., 2013). Additionally, *TaEXPA2* was shown to be induced by high salinity in wheat, and its overexpression enhanced salt stress tolerance in transgenic plants (Chen et al., 2018). In barley, *HvEXPB7* was predominantly expressed in roots, and *HvEXPB7* silencing significantly suppressed root hairs, leading to reduced potassium content under drought conditions (He et al., 2015).

Genomic analysis has shown that there is a total of 75 expansins in soybean. The first identified expansin gene was *GmEXP1*, which is an α-expansin gene that is expressed in roots. Its overexpression accelerated cell enlargement and root growth in transgenic tobacco lines (Lee et al., 2003). *GmEXPB2*, which is a β-expansin gene, was cloned from a P_i_ starvation-induced soybean cDNA library. It was primarily expressed in roots and was induced by P_i_ starvation. *GmEXPB2* overexpression improved hairy root elongation and subsequently affected plant growth and P absorption (Guo et al., 2011; Zhou et al., 2014). To date, although there are 15 EXLB genes in the soybean genome (Zhu et al., 2014), their function has not been confirmed with experimental evidence. We analyzed the expression of expansin genes in the transcript profiles in the public database (Libault et al., 2010) and our own dataset on soybean, and identified a gene (*Glyma. 17 g147500*) that was highly expressed in the root, especially at LP conditions. We hypothesized that this gene might play an important role in plant response to P deficiency. To test this hypothesis, we cloned the corresponding gene, named *GmEXLB1*, from ZH15 (high-P efficiency soybean genotype) and examined its biological function in *Arabidopsis*. The results suggest that the gene may play a role in regulating root architecture to enhance P acquisition.

## Materials And Methods

### Plant Materials and Growth Conditions

The soybean cultivar zhonghuang15 (ZH15, high-P efficiency) and *Arabidopsis* (Columbia ecotype) were used in this study. The normal phosphorus (NP) and low phosphorus (LP) treatments in this study were conducted using a modified Hoagland solution with either 1 mmol/L KH_2_PO_4_ or 1 mmol/L phytate that was adjusted to a pH of 6.0.

### Quantitative RT-PCR

The ZH15 seeds were placed in pots with vermiculite in a greenhouse and grown under a 12-h light (28°C)/12-h dark (24°C) cycle. After 7 days (d) of growth (0 day served as a control), the seedlings were separately treated with NP and LP. The roots were sampled after 7, 14, 21, 28, 35, 42, 49, 56, 63, and 70 days and were used for temporal gene expression profiling.

Total RNA was extracted using an RNAprep Pure Plant Kit (Tiangen, China). Then, the first-strand cDNA was synthesized with a PrimeScriptTM Reagent kit and gDNA Eraser (Takara, Japan). Quantitative RT-PCR (qPCR) was performed with the EvaGreen® qPCR Master Mix (US Everbright® Inc., USA) on a CFX96 Real-Time PCR Detection System (Bio-Rad, USA). Primers for *GmPAP14* (5′-TCAAGCAGCCCCTTCATTAG-3′ and 5′-AGTTTTCCTTCGGCAATCTTC-3′) and the housekeeping gene *GmActin11* (5′-ATCTTGACTGAGCGTGGTTATTCC-3′ and 5′-GCTGGTCCTGGCTGTCTCC-3′) were used for qPCR. The relative expression was calculated using the 2^−ΔΔCt^ method (Livak and Schmittgen, 2001). Three technical replicates were performed for all PCR samples.

### Vector Construction and Plant Transformation

To analyze gene function, the full-length cDNA of *GmEXLB1* was cloned and ligated into the pCHAC vector containing a HA tag. The primers used for cloning were as follows: 5′-GTCGACATGGAGCTTAGTTTTAAGCAC-3′ (*Sal*I site underlined) and 5′-GGATCCTTTAAGCTGAACCTTGGTG-3′ (*BamH*I site underlined). To construct the promoter vector, a 2212-bp *GmEXLB1* promoter sequence was amplified by PCR with the primers 5′-AAGCTTTTGGTAATCAACAAATACATCATC-3′ (*Hind*III site underlined) and 5′-GGATCCGTCTAAATGACAAATTAAATTCTC-3′ (*BamH*I site underlined). This amplified fragment was inserted into the *Hind*III and *BamH*I sites in the pCamG vector. Subsequently, these vectors were transferred into *Agrobacterium tumefaciens* GV3101, and the transgenic plants were obtained by an *Agrobacterium*-mediated floral dip (Clough and Bent, 1998).

### β-Glucuronidase (GUS) Histochemical Staining

To further analyze the *GmEXLB1* expression pattern, the T_3_ *GmEXLB1* promoter-*GUS* (*GmEXLB1::GUS*) transgenic plants were grown under NP conditions, and the different tissues (geminated seed, 3-days seedling, 7-days seedling, flower, pollen grain, and immature pod) were harvested for GUS staining.

To study the involvement of *GmEXLB1* in lateral roots in response to low P, *GmEXLB1::GUS* transgenic plants were grown on agar under NP and LP conditions. Then, the roots were harvested for GUS staining after 12 days. The GUS staining was performed described as (Kong et al., 2018). The samples were incubated in 2 mL tube at 37°C for 3 h in GUS staining buffer (2 mM 5-bromo-4-chloro-3-indolyl-bglucuronic acid in 50 mM sodium Pi buffer, pH 7.2) containing 0.1% Triton X-100, 2 mM K_4_Fe(CN)_6_, 2 mM K_3_Fe(CN)_6_, and 10 mM EDTA). Then, the stained samples were observed and imaged using a BX51 microscope (Olympus, Japan).

### Immunoblotting Analysis of GmEXLB1

Western blot analysis was performed as described in Wang et al. (2011). The total protein was extracted by using the Plant Protein Extraction Kit (CWBIO, China) from 20-days-old seedlings grown in NP conditions. The total protein (60 μg) was separated on a 12% SDS-polyacrylamide gel and transferred to a nitrocellulose filter membrane (Amersham, USA). The membrane was blocked with 5% milk in phosphate-buffered saline. The primary antibody [HA Tag monoclonal antibody (1:5000, Thermo Fisher, United States)] and the secondary antibody [goat anti-mouse IgG-HRP (1:3000, Sangon, China)] were used for the Western blot. The blotted membrane was incubated with ECL luminous solution MaxiLumin-WB (Biokits tech Inc., China) and visualized using an Odyssey FC imaging system (LI-COR, United States).

### Root Trait Measurements in Transgenic Plants

Wild-type and *GmEXLB1*-overexpressing plants were sterilized and plated on agar solid medium containing NP or LP in 13 × 13 petri dishes. After 15 days, the seedlings were used for measuring root traits. The macrographs were obtained by a D7200 camera (Nikon, Japan), and the microscopic images were obtained using a BX51 microscope (Olympus, Japan). ImageJ was used to analyze the images.

### P Content Measurement

The P content was measured by a modified method (Lu et al., 2016). Wild-type and transgenic plants overexpressing *GmEXLB1* were sown on vermiculite. NP and LP were supplied to the seedlings. The shoots were harvested after 30 days of treatment. Then, the samples were dried at 80°C and digested in glass tubes with H_2_SO_4_ and H_2_O_2_ at 180°C. The digested solutions were incubated with malachite green reagent at room temperature and measured at 650 nm.

### Statistical Analysis

All data were analyzed using SPSS 17.0 software (IBM, United States). One-way ANOVA with an LSD at *p* < 0.05 was used to identify the differences between the observations.

## Results

### Cloning and Bioinformational Assessment of *GmEXLB1* in Soybean

**Figure 1:**
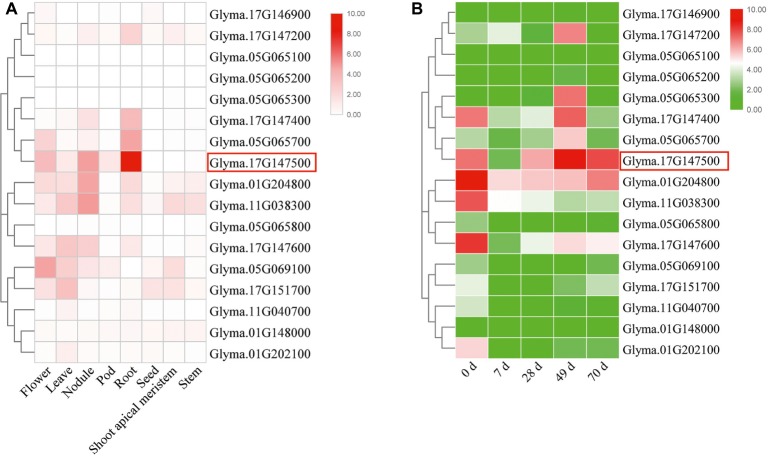
Expression analysis of GmEXLBs. **(A)** Expression of GmEXLBs in different tissues/organs. RNA-seq data (Libault et al., 2010) are shown as a heat map. The color scheme used to represent the expression level is white/red: white indicates a low variation in expression and red indicates a high variation in expression. **(B)** Expression of GmEXLBs in roots. RNA-seq data from ZH15 roots treated with low phosphorus (LP) are shown as a heat map. The color scheme used to represent the expression level is green/red: green indicates a low variation in expression, and red indicates a high variation in expression.

**Figure 2:**
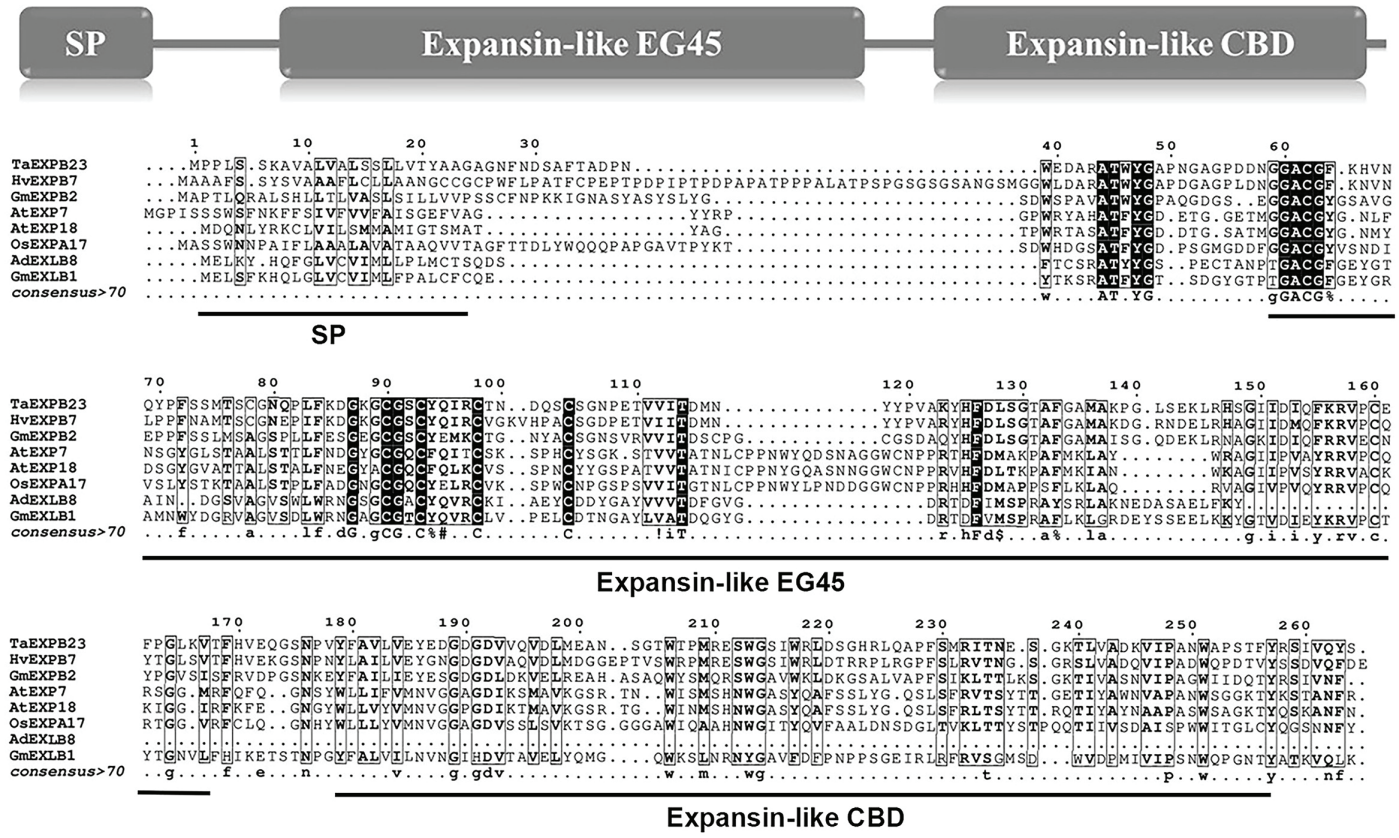
Alignment of the amino acid sequences of GmEXLB1 and other expansins. Alignment of GmEXLB1 with TaEXPB23 (AAP84631.1), HvEXPB7 (ALP44111.1), GmEXPB2 (ACA83732.1), OsEXPA17 (XP_015642083.1), AtEXP7 (NP_172717.1), AtEXP18 (NP_176486.1), and AdEXLB8 (XP_015963649.1). Conserved sequence motifs are indicated by arrows. SP, signal peptide; Expansin-like EG45, expansin, family 45 endoglucanase-like domain; Expansin-like CBD, expansin, cellulose-binding-like domain.

**Figure 3:**
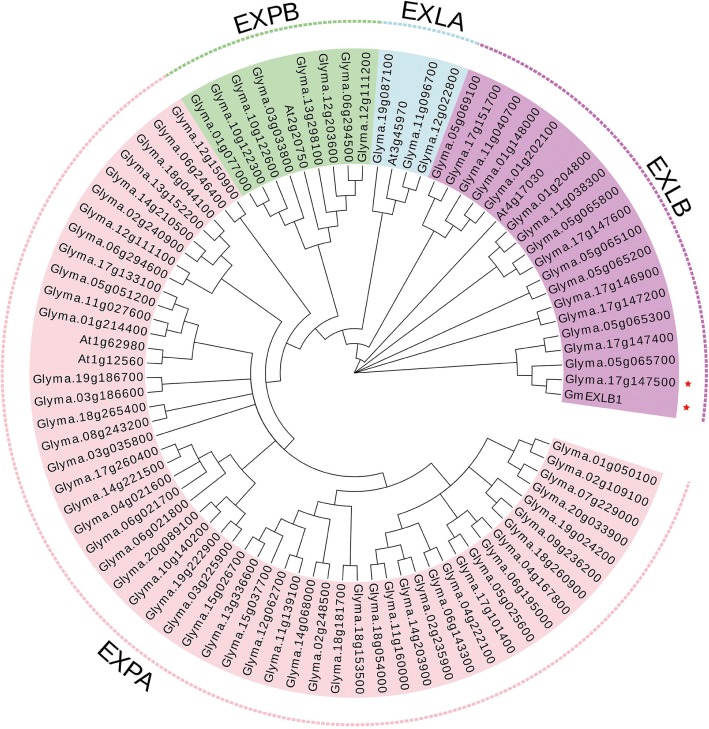
Phylogenetic tree of expansins in soybean and *Arabidopsis*. Using expansins in soybean and *Arabidopsis*, a phylogenetic tree was constructed using the neighbor-joining method with 1,000 bootstraps in MEGA 7.0 and was modified by EvolView. The four expansin subfamilies are: EXPA, α-expansin; EXPB, β-expansin; EXLA, expansin-like A; and EXLB, expansin-like B.

In this study, we first analyzed the transcriptome profiles of GmEXLBs by retrieving the RNA-seq data for soybean. We found that *Glyma.17G147500* was more highly expressed in roots than other GmEXLBs (Figure 1A), and its expression was induced from 28 to 70 days under LP conditions (Figure 1B). Thus, we inferred that *Glyma.17G147500* was an important gene involved in responses to low P in soybean. Subsequently, we cloned the full-length sequence of *Glyma.17G147500* from ZH15, which is referred to as *GmEXLB1*. The length of the *GmEXLB1* cDNA was 744 bp and encoded a polypeptide of 248 amino acid residues. Bioinformatics analysis showed that the molecular mass of GmEXLB1 was approximately 27.67 kDa and that it had an isoelectric point of 6.57. Predication of subcellular location showed that GmEXLB1 was located in extracellular region of cell. The first 24 amino acids of the N terminus in GmEXLB1 were considered a signal peptide (SP). These predications indicated that GmEXLB1 might be a secreted protein. Subsequently, analysis of a multialignment showed that GmEXLB1 shared significant similarity with other expansin proteins in terms of its EG45 and CBD domains (Figure 2). Using expansin genes in soybean and *Arabidopsis*, a phylogenetic tree was constructed by MEGA 7.0 and modified by EvolView. The results indicated that GmEXLB1 was classified into the EXLB group and was highly homogenous with Glyma.17G147500 (Figure 3).

### *GmEXLB1* Was Induced in the Lateral Roots of Soybean Under Low P Conditions

**Figure 4:**
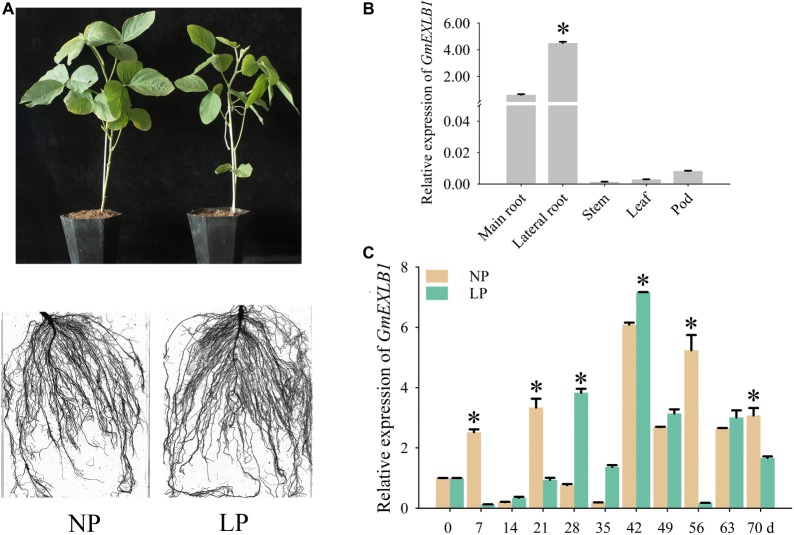
Analysis of *GmEXLB1* expression patterns in soybean. **(A)** Phenotype of zhonghuang15 (ZH15) under normal phosphorus (NP) and low phosphorus (LP) conditions. **(B)** Expression analysis of *GmEXLB1* in different ZH15 tissues under LP conditions. The relative expression was calculated using the 2^−ΔΔCT^ method, and the data are the mean ± SE (*n* = 3). Error bars represent the SE. Asterisks indicate significant differences (*p* < 0.05). **(C)** Expression of *GmEXLB1* in lateral roots in ZH15. Seven-day-old seedlings were treated with NP and LP (0 day seedlings were used as a control). Lateral roots were sampled after 7, 14, 21, 28, 35, 42, 49, 56, 63, and 70 days and were used for temporal expression analysis. The relative expression was calculated using the 2^−ΔΔCT^ method, and the data are the mean ± SE (*n* = 3). Error bars represent the SE. Asterisks indicate significant differences (*p* < 0.05).

To confirm the expression pattern of *GmEXLB1*, we examined the expression of *GmEXLB1* in ZH15 by qPCR (Figure 4). The spatial expression results showed that *GmEXLB1* was mainly expressed in lateral roots in comparison to the main roots, leaves, stems, and pods (Figure 4B). Then, the temporal expression of *GmEXLB1* in the lateral roots of soybean revealed that *GmEXLB1* was expressed at lower levels on day 7 and 21 compared to NP plants but was expressed at higher level from day 28 to 42 (Figure 4C). The above results suggest that *GmEXLB1* is a phosphate starvation-induced gene in the lateral roots of soybean.

### Histochemical Observation of the *GmEXLB1* Promoter in Transgenic *Arabidopsis*

**Figure 5:**
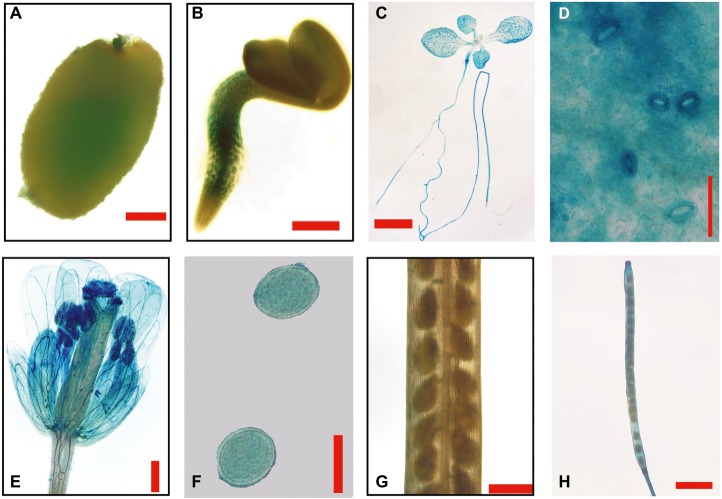
GUS staining in different tissues of transgenic *Arabidopsis* with *GmEXLB1::GUS*. **(A)** Germinating seed. The scale bar = 100 μm. **(B)** Three-day-old seedlings. The scale bar = 200 μm. **(C)** Seven-day-old seedlings. The scale bar = 2 cm **(D)** Stomata. The scale bar = 50 μm. **(E)** Mature flower. The scale bar = 500 μm. **(F)** Pollen grain. The scale bar = 20 μm. **(G)** Immature seeds. The scale bar = 500 μm. **(H)** Immature pod. The scale bar = 2 cm.

To study the regulation of the *GmEXLB1* promoter, a 2,200-bp promoter sequence from the gene was fused to a GUS reporter gene (*GmEXLB1::GUS*), which was transferred into wild-type *Arabidopsis* plants. Initially, we analyzed GUS histochemical staining in different tissues. The results showed that GUS staining was detected in germinated seeds (Figure 5A) and the root in 3-day seedlings (Figure 5B). As the seedlings grew, GUS staining was observed in the root system (Figure 5C), leaves (Figures 5C,D), and guard cells (Figure 5D). At the reproductive stage, GUS staining was also observed in mature flowers (Figure 5E), pollen grains (Figure 5F), and immature pods (Figure 5H), not in immature seed (Figure 5G).

**Figure 6:**
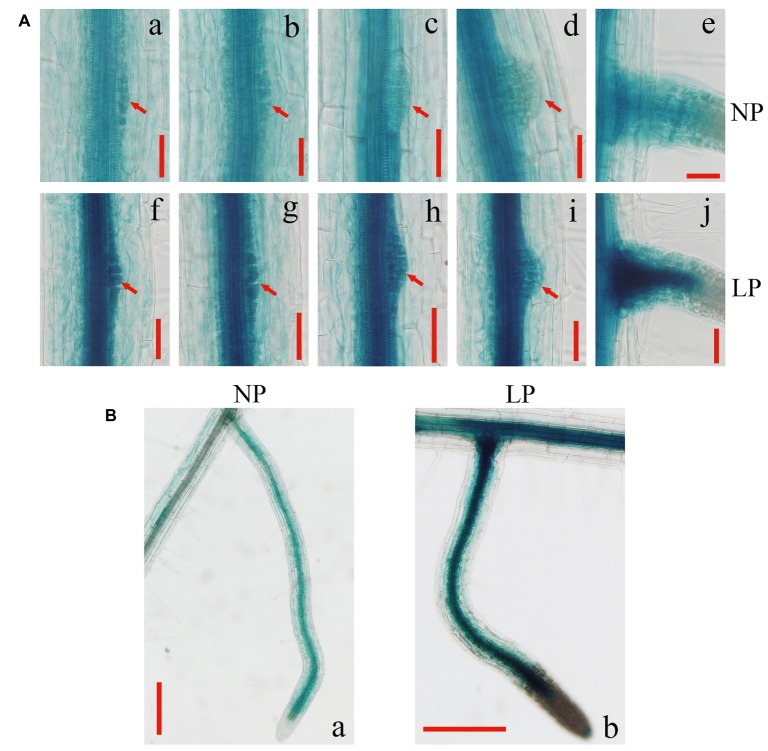
Expression patterns of *GmEXLB1::GUS* during lateral root development. **(A)** **(a–e)** GUS staining at different stages of lateral root emergence in transgenic Aradidopsis under NP conditions **(f–j)** GUS staining at different stages of lateral root emergence in transgenic Aradidopsiss under LP condition. Red arrows indicate primordium margins. The scale bar = 50 μm. **(B)** GUS staining of mature lateral roots in transgenic *Arabidopsis* under NP and LP conditions. The scale bar = 200 μm.

To test whether the induced expression of *GmEXLB1* is involved in lateral root development, we analyzed the patterns of *GmEXLB1::GUS* signals in the roots of transgenic *Arabidopsis* at the different developmental stages of lateral root formation (Figure 6). Under LP conditions, stronger GUS staining was observed in the stele of roots and in lateral root primordium cells (Figures 6Ad–Aj) compared with that under NP conditions (Figures 6Aa–Ae). During maturation of the lateral root, GUS signals were located in vascular bundles in the elongation and maturation zones (Figure 6Ba). By contrast, GUS staining was increased under LP conditions (Figure 6Bb). These results suggested that the expression of *GmEXLB1* was associated with the development of lateral roots.

### *GmEXLB1* Overexpression Altered Root Architecture in *Arabidopsis* Under Low P Conditions

**Figure 7:**
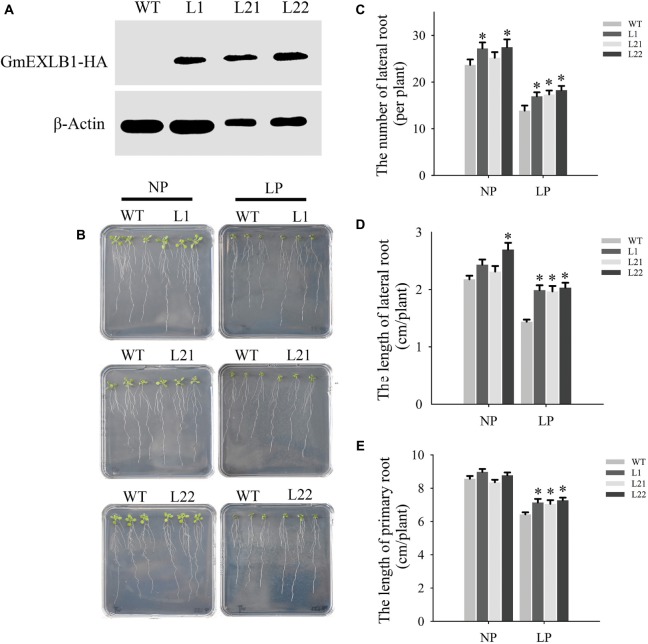
Root phenotypic analysis of transgenic *Arabidopsis* with *GmEXLB1* overexpression. **(A)** Western blotting of transgenic and wild-type plants. WT: wild-type plant. L1, L21, and L22: three transgenic lines. **(B)** Root phenotype observed on agar with NP and LP. **(C)** The number of lateral roots in transgenic and wild-type plants under NP and LP conditions. The data are the mean ± SE (*n* = 5). Error bars represent the SE. Asterisks indicate that the values are significantly different (*p* < 0.05). **(D)** The lateral root lengths in transgenic and wild-type plants under NP and LP conditions. The data are the mean ± SE (*n* = 5). Error bars represent the SE. Asterisks indicate that the values are significantly different (*p* < 0.05). **(E)** The primary root length in transgenic and wild-type plants under NP and LP conditions. The data are the mean ± SE (*n* = 5). Error bars represent the SE. Asterisks indicate that the values are significantly different (*p* < 0.05).

To evaluate whether *GmEXLB1* regulates the root system, we constructed a *GmEXLB1* gene that was driven by the *CaMV 35S* promoter and added an HA tag at its C terminal. Subsequently, this *GmEXLB1* construct was transferred into *Arabidopsis* by using an *Agrobacterium*-mediated method (Clough and Bent, 1998). Bioinformatic analysis suggested that GmEXLB1 might be a soluble protein and located in extracellular region of cell. So, the total proteins of three independent T_3_ transgenic lines (L1, L21, and L22) were detected by Western blotting (Figure 7A). Then, we investigated how *GmEXLB1* influences root system architecture. The transgenic and wild-type plants were grown on modified MS medium with NP or LP. Fifteen days after treatment, the root phenotype was observed (Figure 7B) and measured (Figures 7C–E). Under LP conditions, compared to wild-type plants, the transgenic lines overexpressing *GmEXLB1* had greater numbers of lateral roots with increases of 22.11% (L1), 29.32% (L21), and 31.73% (L22) (Figure 7C) and longer lateral roots with increases of 38.9% (L1), 38.06% (L21), and 41.28% (L22) (Figure 7D). Additionally, these characteristics improved under NP conditions in some transgenic lines compared to the wild type. In addition, L1, L21, and L22 exhibited longer primary roots with increases of 14.0813.59, and 18.11%, respectively, compared to those of the wild type under LP conditions (Figure 7E).

### *GmEXLB1* Overexpression Promoted Cell Elongation in *Arabidopsis* Roots Under Low P Conditions

**Figure 8:**
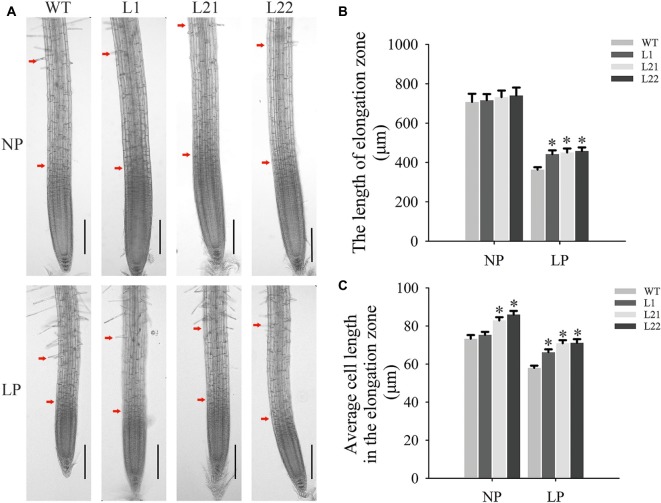
Microscopic observation and measurements in the elongation zone of transgenic plants. **(A)** Microscopic observation of transgenic and wild-type plants under NP and LP conditions. The distance between the two red arrows represents the elongation zone. The scale bar = 200 μm. **(B)** The elongation zone length in transgenic and wild-type plants under the NP and LP conditions. The data are the mean ± SE (*n* = 5). Error bars represent the SE. Asterisks indicate that the values are significantly different (*p* < 0.05). **(C)** The cell length in the elongation zone in transgenic and wild-type plants under NP and LP conditions. The data are the mean ± SE (*n* = 150). Error bars represent the SE. Asterisks indicate that the values are significantly different (*p* < 0.05).

To explore the influence of changes in root architecture on transgenic *Arabidopsis*, microscopic observations and measurements of roots were performed in this study (Figure 8A). The results showed that *GmEXLB1* overexpression significantly increased the length of the elongation zone (Figure 8B) with increases of 21.71 (L1), 24.17 (L21), and 26.18% (L22) and the average cell length in the elongation zone (Figure 8C) with increases of 14.18 (L1), 22.29 (L21), and 22.72% (L22) in plants under LP conditions compared to wild-type plants.

### *GmEXLB1* Overexpression Improved P Acquisition in *Arabidopsis* Under Low P Conditions

**Figure 9:**
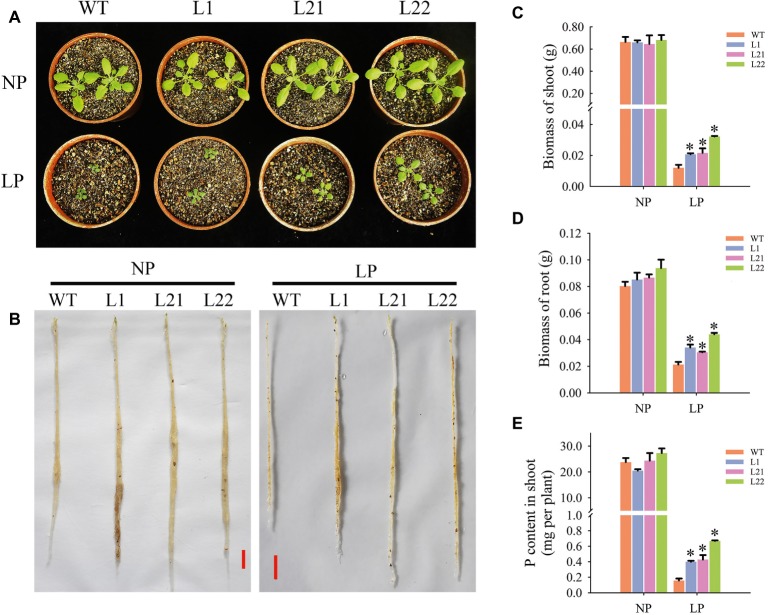
Measurements of P acquisition efficiency in transgenic plants under different phosphorus conditions. **(A)** Phenotypic observation of shoots under NP and LP conditions. **(B)** Phenotypic observation of roots under NP and LP conditions. The scale bar = 1 cm. **(C)** Biomass of shoots measured in transgenic and wild-type (WT) plants under NP and LP conditions. The data are the mean ± SE (*n* = 5). Error bars represent the SE. Asterisks indicate that the values are significantly different (*p* < 0.05). **(D)** Biomass of roots measured in transgenic and wild-type (WT) plants under NP and LP conditions. The data are the mean ± SE (*n* = 5). Error bars represent the SE. Asterisks indicate that the values are significantly different (*p* < 0.05). **(E)** P content of shoots measured in transgenic and wild-type (WT) plants under NP and LP conditions. The data are the mean ± SE (*n* = 5). Error bars represent the SE. Asterisks indicate that the values are significantly different (*p* < 0.05).

Changes in root architecture influence P acquisition and utilization in plants. In this study, under LP conditions, transgenic plant growth was improved compared with that of the wild-type plants (Figures 9A,B), as the weights of fresh shoots increased by 72.5 (L1), 80.0 (L21), and 168.33% (L22) (Figure 9C) and the weights of fresh roots increased by 60.09 (L1), 43.19 (L21), and 106.57% (L22) (Figure 9D). In addition, the P contents in shoots from the three transgenic lines significantly increased by 152.0 (L1), 170.86 (L21), and 322.98% (L22) (Figure 9E). These results indicated that *GmEXLB1* overexpression enhanced P acquisition efficiency by changing root architecture.

## Discussion

Expansins are ubiquitous in plants and comprise a superfamily that is classified into four subfamilies: EXPA, EXPB, EXLA, and EXLB (Sampedro and Cosgrove, 2005). To date, few EXLA and EXLB genes have been studied (Cosgrove, 2015), but EXPA and EXPB genes have been identified *via* experimental evidence in *Arabidopsis* and rice (Lin et al., 2011; Zhang et al., 2011; Lee and Kim, 2013; Che et al., 2016). P is a necessary macronutrient, and different EXPA and EXPB gene expression patterns under low P conditions have been found in plants, including soybean. However, the expression of EXLB genes in response to low P has not been elucidated. In this study, we examined *GmEXLB1* gene for its function. Our data suggested that the gene may play an important role in plant response to LP conditions (Figure 1). This suggested that these genes may have a certain ability to help soybean cope with low P-induced stress. Based on these results, we chose and cloned the gene named *GmEXLB1* in Zh15 using the sequence of *Glyma.17G147500*. Under low P conditions, the induced expression of *GmEXLB1* in lateral roots suggested that it might play an important role in the response of roots to P starvation.

Previous studies have reported that expansin genes are very important for root growth and development (Li et al., 2003, 2011; Zhao et al., 2012; Wang et al., 2014). For example, *OsEXPA10* is expressed in roots, and knockout of the gene resulted in a significant decrease in cell elongation in the root in the absence of aluminum (Che et al., 2016). In our paper, GUS staining in roots was mainly observed in the stele and lateral root primordium cells (Figure 6). As pericycle cells in the stele are specialized cells that form lateral roots (Lopez-Bucio et al., 2005), this result indicated that *GmEXLB1* may function in lateral root formation. Furthermore, *GmEXLB1* overexpression increased the number of lateral roots in *Arabidopsis* (Figure 7C). A recent study reported that expansin proteins are important for lateral root initiation by Ramakrishna et al. (2019) and *GmEXLB1* may play a similar role in soybean under LP conditions. Additionally, we found that the overexpression of the gene improved the length of the lateral root of *Arabidopsis* (Figure 7D). Other studies have reported that root growth and development are affected by root apical meristem elongation (Perilli et al., 2012; Alarcón et al., 2014). Here, we found that the elongation zone and average cell length in the elongation zone in transgenic *Arabidopsis* was significantly increased (Figure 8), which might explain how *GmEXLB1* could promote lateral root length. Similar results were reported that showed that *GmEXP1* was involved in the elongation and/or the initiation of primary and secondary roots (Lee et al., 2003) and that *GmEXPB2* increased root cell division and elongation to enhance lateral root length in *Arabidopsis* (Guo et al., 2011). Thus, these genes have similar functions in root development, although they are classified into different subfamilies. Based on the fact the average increase in cell length in the elongation zone is similar to the average increase in the length of the elongation zone, it appears that GmEXLB1 is only involved in regulation of cell elongation and not in cell division. This notion is supported by the fact that the gene shows little expression in the very tip of the root or in the cell division region based on the GUS staining assay.

It is well known that changes in root architecture play a critical role in allowing plants to cope with a variety of abiotic stresses (Rellan-Alvarez et al., 2016). For example, the overexpression of *TaEXPB23* enhanced water stress tolerance in tobacco by facilitating the lengthening of the lateral root (Li et al., 2015). Although many expansin genes have been shown to be involved in the alteration of the root system to increase tolerance to abiotic stresses, only a few studies have reported that expansins are increased in response to low P. For instance, microarray analysis has indicated that expansin genes are responsive to P_i_ levels in *Arabidopsis*; however, more in-depth studies have not been reported (Morcuende et al., 2007). Han et al. (2014) found that some expansins (five *EXPAs* and nine *EXPBs*) were up- or down-regulated in response to P in wheat. The overexpression of *TaEXPB23* increased the number of lateral roots in wheat under low P conditions (Han et al., 2014). In soybean, it was first reported that *GmEXPB2* overexpression enhanced *Arabidopsis* growth and P uptake at low P levels (Guo et al., 2011; Zhou et al., 2014).

In our present work, the transgenic plants with *GmEXLB1* had improved growth compared with that of the wild type (Figures 9A,B). Additionally, in contrast to the wild type, transgenic *Arabidopsis* had more biomass and P content in shoots under LP conditions (Figures 9C–E). P deficiency severely limits crop yields, and more fertilizer applications are required to obtain high yields. However, enhancing P fertilizer use in agricultural systems and developing plants with P use efficiency are necessary to reduce the use of P fertilizer and to prevent soil degradation (Heuer et al., 2017). Compared to those of the wild type, greater biomass and P content were detected in transgenic *Arabidopsis* that overexpressed *GmEXLB1* under LP conditions, but enhancement in P use efficiency was not found (data not shown). Phospholipid metabolism (Pant et al., 2015) and the remobilization of P_i_ from vacuoles (Xu et al., 2019) are important approaches to improve internal P use efficiency. It is possible that more external P uptake that alleviated P deficiency suppressed the remobilization of internal P in transgenic *Arabidopsis* under LP conditions. Overall, we showed that *GmEXLB1* overexpression enhanced P uptake by facilitating lateral root development.

It is interesting that the gene is also expressed in leaf, highly in guard cells, and in reproductive organs as well as in the vascular tissues of the root based on GUS staining assay. The data suggest *GmEXLB1* may play other functions in plant growth and development. Other studies have reported that expansin genes affect the development of other organs or tissues besides roots, including leaves (Madoka Gray-Mitsumune et al., 2008), grains (Lizana et al., 2010), flowers (Harada et al., 2011), fruit (Perini et al., 2017), and guard cells (Zhang et al., 2011). More studies are needed to address other functions of this gene.

In summary, we identified an important EXLB gene, *GmEXLB1*, in soybean. Our data indicate that *GmEXLB1* is closely associated with lateral root emergence and root elongation. When P is insufficient, *GmEXLB1* is induced and produces changes in root morphology to enhance the absorption of P.

## Data Availability

Publicly available datasets were analyzed in this study. This data can be found here: https://phytozome.jgi.doe.gov/pz/portal.html.

## Author Contributions

CZ, YK, and BW conceived of and designed the experiments. YK and BW participated in the entirety of the experiment and analyzed the data. YK drafted the manuscript. HD and WL provided suggestions during the experiments. All authors participated in the revision of the manuscript.

### Conflict of Interest Statement

The authors declare that the research was conducted in the absence of any commercial or financial relationships that could be construed as a potential conflict of interest.
